# Mobile medial pivot (lateral slide) type total knee arthroplasty exhibits a medial pivot pattern: three-dimensional motion analysis using cadaveric knees

**DOI:** 10.1186/s40634-022-00558-9

**Published:** 2022-12-15

**Authors:** Osamu Tanifuji, Tomoharu Mochizuki, Takashi Sato, Satoshi Watanabe, Go Omori, Hiroyuki Kawashima

**Affiliations:** 1 Division of Orthopaedic Surgery, Department of Regenerative and Transplant Medicine, Niigata University Graduate School of Medicine and Dental Science, 1-757 Asahimachi-Dori Chuo-Ku, Niigata, 951-8510 Japan; 2 Department of Orthopaedic Surgery, Niigata Medical Center, Niigata, Japan; 3 Department of Health and Sports, Faculty of Health Sciences, Niigata University of Health and Welfare, Niigata, Japan

**Keywords:** Total knee arthroplasty, Knee kinematics, Mobile-bearing, Medial pivot, Three-dimensional to two-dimensional registration technique

## Abstract

**Purpose:**

The purpose of this study was to analyze the dynamic kinematics of the mobile medial pivot-type total knee arthroplasty (MMPTKA) using the three-dimensional (3D)-to-2D registration technique.

**Methods:**

Cadaveric knees from five humans were used. Computed tomography of the lower limb and preoperative 3D planning for MMPTKA were performed. After performing TKA, passive motion of the knee was observed from a fully extended position to maximum flexion using a flat panel detector. The following parameters were determined: (1) anteroposterior (AP) translations of the medial and lateral most distal points (estimated contact point) of the femoral component, (2) rotational femoral component’s X-axis (FCX) angle, and (3) rotational insert angle. Paired *t-*tests were used to analyze differences in the AP translation between the medial and lateral most distal points of the femoral component as well as differences in the changes in the rotational angle between the FCX and X-axis of the insert on the tibial component’s axial plane.

**Results:**

The AP translations of the femoral component’s medial and lateral most distal points were 8.4 ± 2.5 and 13.6 ± 3.3 mm, respectively (*p* = 0.001). The rotational angles of the FCX and insert were 10.7° ± 4.9° external rotation and 8.9° ± 4.1° internal rotation, respectively (*p* = 0.004).

**Conclusions:**

The posterior translation of the lateral side of the femoral component was greater than that of the medial in all cases. Hence, a medial pivot pattern was identified. The femoral component exhibited external rotation throughout knee flexion in all subjects, whereas the mobile insert exhibited internal rotation (opposite pattern relative to the femoral component). This study provides valuable kinematical information of MMPTKA that has not been clear yet.

## Introduction

Total knee arthroplasty (TKA) is the gold standard for treating patients with end-stage knee osteoarthritis (OA), and implants of various designs have been developed to improve clinical outcomes and patient satisfaction. Mobile-bearing-type TKA (MBTKA) was designed in the late 1970s, and the reduction of polyethylene wear and a self-alignment mechanism were expected as a design concept [6, 29, 30]. Several MBTKA kinematic studies have demonstrated that the femoral component is externally rotated relative to the tibial component in the standing position and that the rotation of the femoral component is guided by the rotation of the mobile-bearing insert [7, 10, 11]. In addition, long-term clinical outcomes have been reported using MBTKA, and good long-term follow-up results over more than 15 years have been demonstrated, with 95% survival [16, 21, 28]. Medial pivot-type TKA (MPTKA) utilizes different types of implants that are designed to reproduce the medial pivot motion observed in the normal knee with a highly medially constrained insert [2, 17, 20]. Several studies on MPTKA kinematics and clinical outcomes demonstrated good results with higher sagittal stability and better patient-reported outcomes [9, 12].

In recent years, TKA, which has both a medial pivot mechanism with high medial constraint and a mobile mechanism of insert, has been developed and clinically used. However, previous studies have only described kinematics of up to less than 90° of the knee joint [3, 4] and the dynamic kinematics of this mobile medial pivot-type TKA (MMPTKA) in the deep flexion phase remains unclear. Whether MMPTKA, which employs both MBTKA and MPTKA mechanisms, can demonstrate both advantages at the same time also remains unclear. Thus, this study aimed to analyze the dynamic kinematics of MMPTKA from full extension to the deep flexion phase using the three-dimensional (3D)-to-2D registration technique.

It was hypothesized that the kinematics of MMPTKA differs from that of previously reported MBTKA and MPTKA.

## Materials and methods

This cadaveric study was performed according to a protocol approved by the investigational review board of our institution (2017–0156). In this study, five human cadaveric knees from patients who had undergone thigh amputation due to lower leg gangrene without poor soft tissue conditions around the knee including skin, obvious medial and lateral collateral ligament deficiency, and without valgus deformity of the knee were used.

### Preoperative planning and TKA

At first, a lower limb computed tomography (CT) scan was obtained for each subject using SOMATOM Sensation 16 (Siemens, Munich, Germany) with a 1-mm interval. Data from the CT scan were used to build a 3D digital model of the bones using the ZedView (LEXI, Tokyo, Japan) visualization and modeling software [23]. Preoperative 3D planning was performed by reading the implant computer-aided design data of Genus MB® (Adler Ortho, Milano, Italy) for all subjects. This implant has both a medial pivot mechanism with high medial constraint and a mobile mechanism of insert. Because the exact mechanical axis of an amputated limb could not be defined, the femoral components were replaced parallel to the surgical epicondylar axis both in the coronal and axial planes during preoperative planning. In the coronal plane, a few degrees of fine-tuning were sometimes performed with reference to the shapes of the femoral posterior condyles. The tibial components were replaced perpendicular to the tibial anatomical axis. Posterior slopes were parallel to the lateral tibial plateau joint surface. Rotational alignments were matched to the line connecting the posterior cruciate ligament insertion and medial border to one-third of the tibial tubercle [1]. All surgeries were performed by a single orthopedic surgeon (OT). TKA was performed using the medial parapatellar approach and the measured resection technique, and attention was paid to maintain the soft tissue balance to avoid medial instability in all ranges of motion. The posterior cruciate ligament was preserved. Regarding the mobile insert, asymmetrically designed inserts (lateral sliding version) were applied with a highly constrained surface on the medial side, and three tantalum markers were embedded into the insert. The wound was closed using 0 monofilament absorbable thread for the joint capsule and 3–0 nylon thread for the skin.

### Motion analysis using a single-plane flat panel detector

**Fig. 1:**
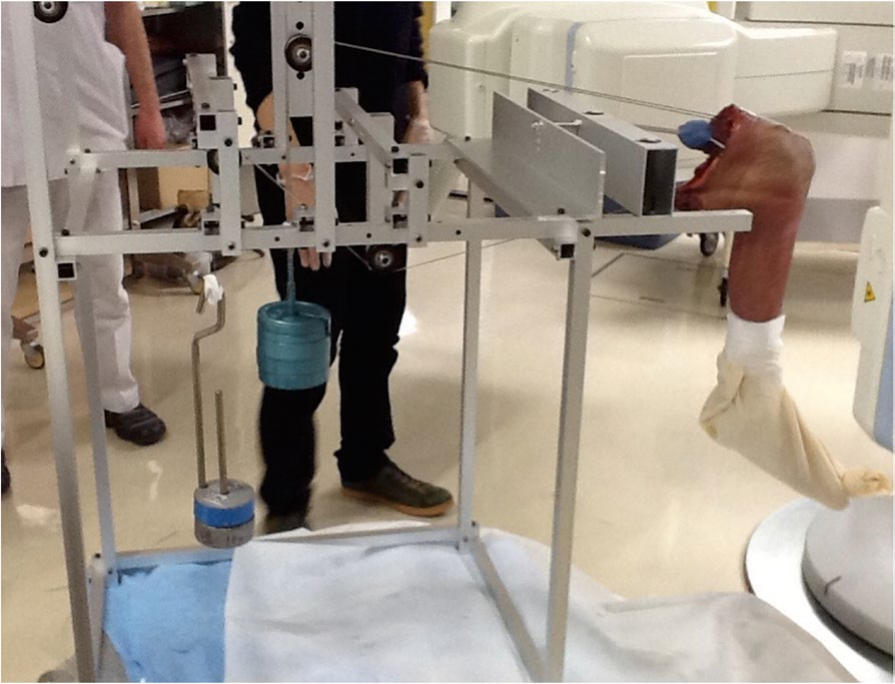
Appearance of the experimental special device. The amputated limb that underwent TKA was fixed to it. Tensile forces of 4 and 2 kg were applied to quadriceps and hamstring muscles, respectively, as the bearing load

The amputated limb was fixed to a special device after performing TKA. This special device was designed to apply a constant tensile force to the quadriceps and hamstring muscles via pulleys (Fig. 1). Then, passive motion of the knee was observed from a fully extended position to maximum flexion and recorded using a flat panel detector (AXIOM Artis® dTA; Siemens). Tensile forces of 4 and 2 kg were applied to the quadriceps and hamstring muscles as the bearing load, respectively. The sampling frequency was 15 Hz, with an image area of 380 × 300 mm and a resolution of 1240 × 960 pixels.

### Evaluation using the 3D-to-2D image registration technique

**Fig. 2:**
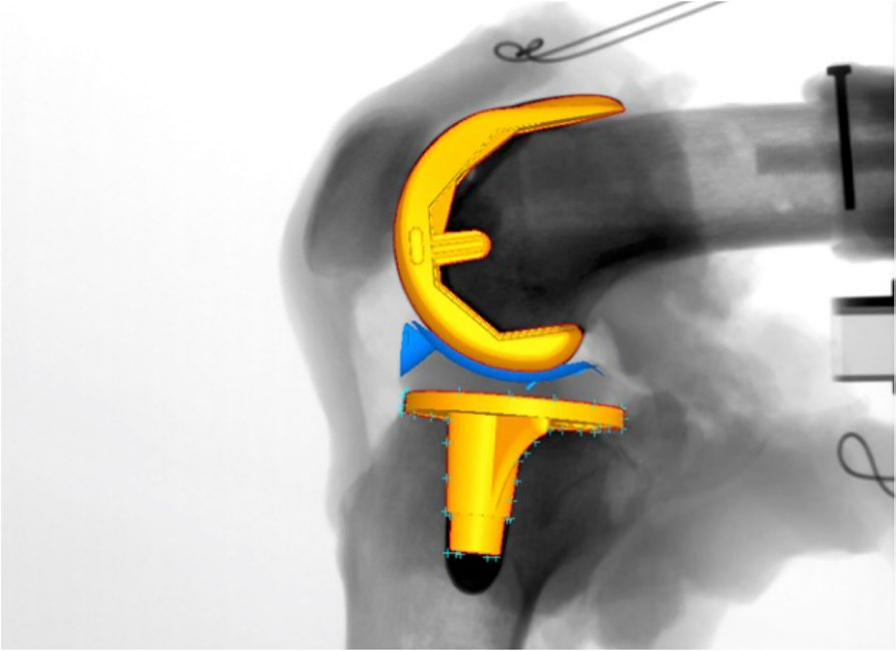
Relative 3D positions of the femoral components and tibial components in each fluoroscopic image were obtained using the 3D-to-2D registration technique. The relative 3D positions of the inserts and tibial components in each fluoroscopic image were obtained using the same method

**Fig. 3:**
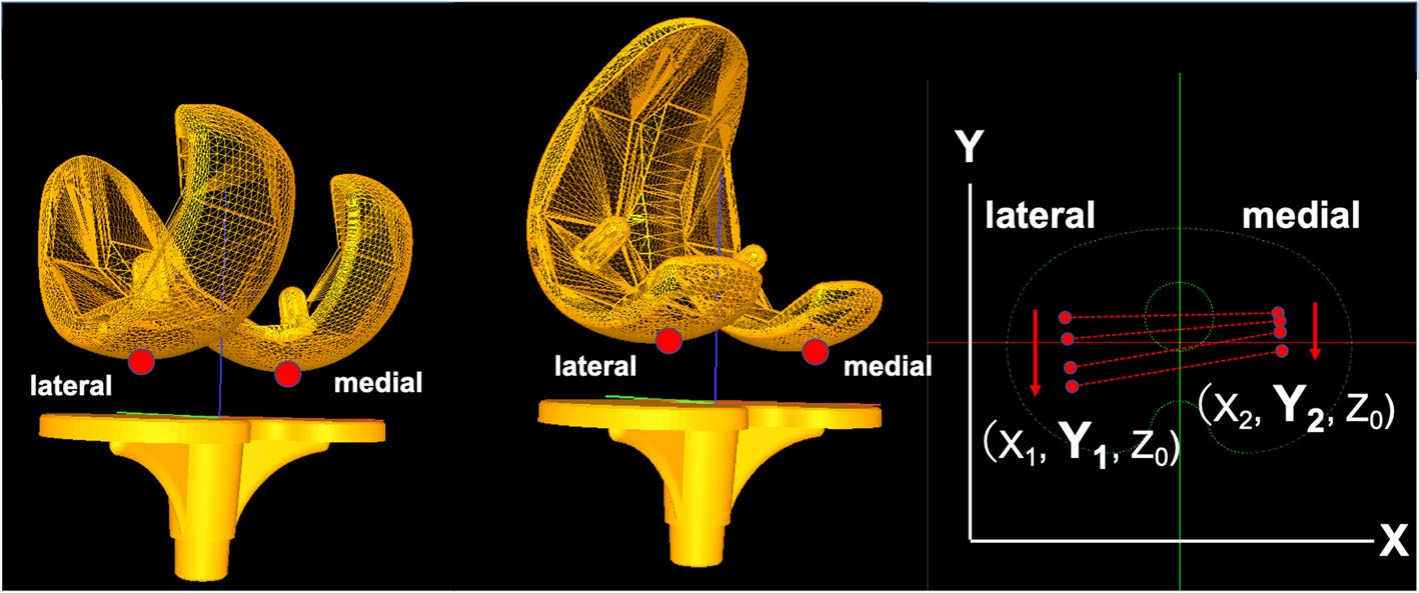
Anteroposterior (AP) translations of the femoral component’s medial and lateral most distal points (estimated contact point) were evaluated as Y-values of the tibial component’s coordinate system

**Fig. 4:**
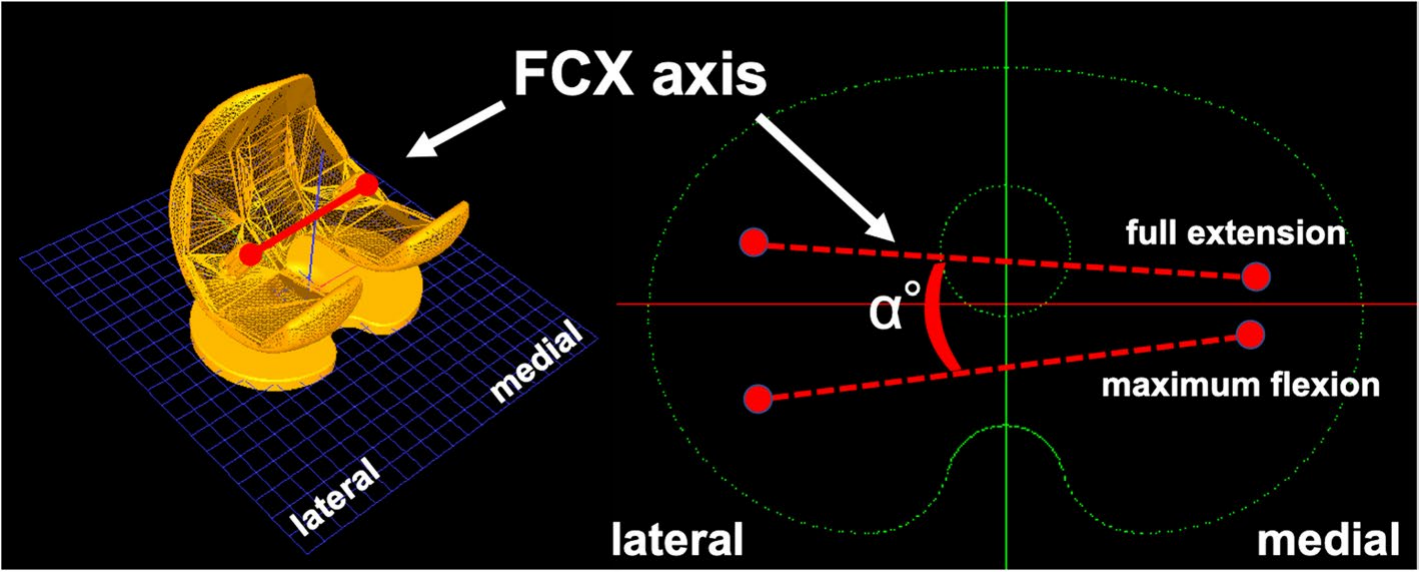
Changes in the femoral component’s X-axis (FCX) angle on the tibial component’s axial plane (rotational angle). The FCX was projected onto the axial (XY) plane of the tibial component’s coordinate system. The rotational angle from full knee extension to maximum flexion was calculated

**Fig. 5:**
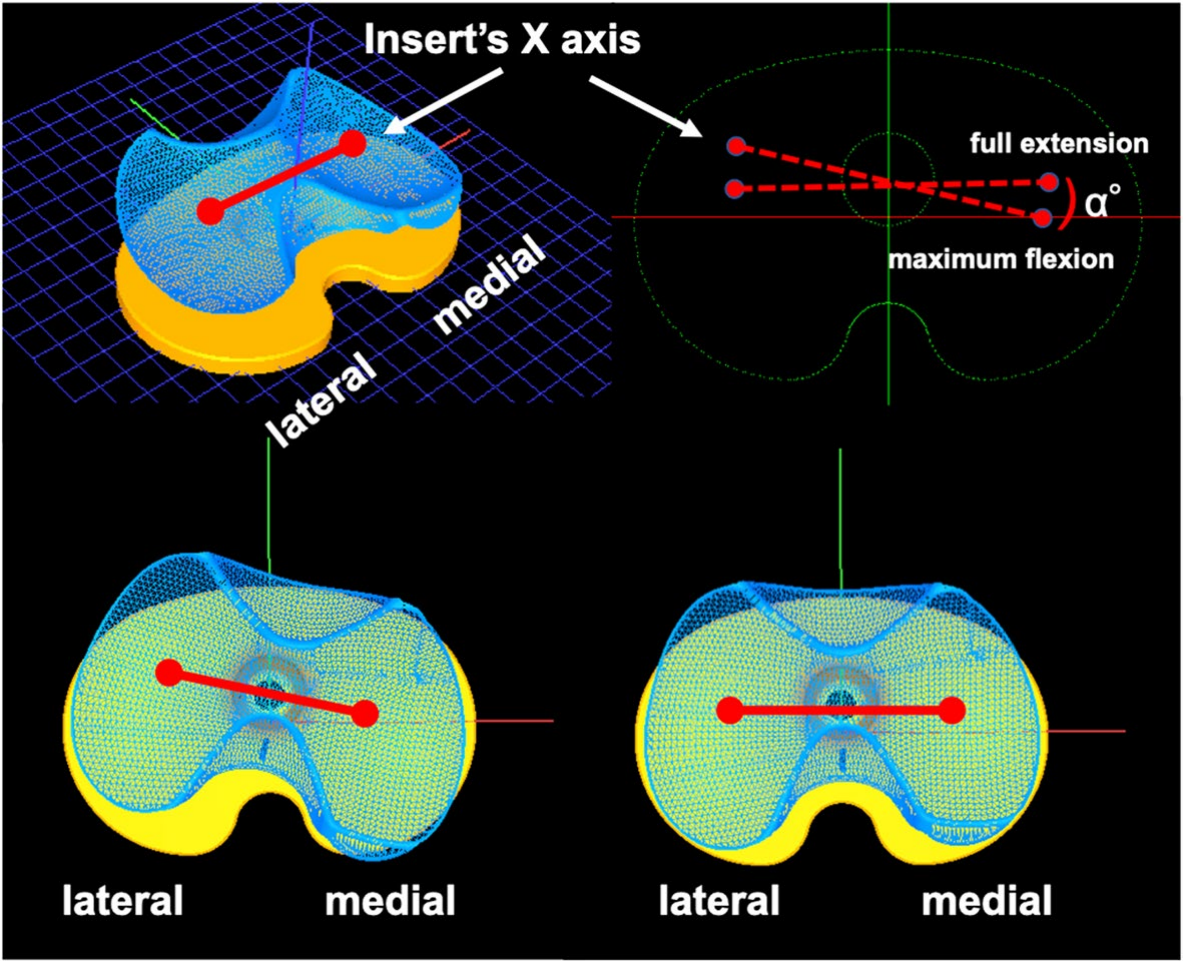
Changes in the angle of the insert’s X-axis on the tibial component’s axial plane (rotational angle). The insert’s X-axis was projected onto the axial (XY) plane of the tibial component’s coordinate system. The rotational angle from full knee extension to maximum flexion was calculated

A series of static lateral images were digitally stored. After manually detecting the femoral and tibial component contours in these images, a 3D-to-2D technique with an automated shape-matching algorithm was employed to determine the relative 3D positions of the femoral and tibial components in each fluoroscopic image (KneeMotion®; LEXI, Tokyo, Japan) (Fig. 2). Relative motion between the femoral and tibial components was determined using this procedure for all images. Similarly, the relative 3D positions of the insert and tibial components in each fluoroscopic image were obtained by matching the tantalum markers of the insert. The root mean square errors were 0.3–0.8 mm for in-plane translation, 2.2 mm for out-of-plane translation, and 0.2°–0.6° for rotation [26, 27]. The anteroposterior (AP) locations of the femoral component’s medial and lateral most distal point (estimated contact point) of all images were evaluated as Y-values of the tibial component’s coordinate system (Fig. 3). Relative motion between the femoral and tibial components was quantified as the movement of the femoral component’s X-axis (FCX) (mediolateral axis) projected onto the axial (XY) plane of the tibial component’s coordinate system (Fig. 4). Relative motion between the insert and tibial component was also quantified as the movement of the insert’s X-axis (mediolateral axis) projected onto the axial (XY) plane of the tibial component’s coordinate system (Fig. 5).

The following parameters were determined: (1) AP translations of the femoral component’s medial and lateral most distal points (estimated contact point), (2) changes in the FCX angle on the tibial component’s axial plane (rotational angle), and (3) changes in the angle of the insert’s X-axis on the tibial component’s axial plane (motion pattern of the insert). AP translation was expressed as the difference between the maximum and minimum values throughout knee flexion. Changes in the FCX angle and the angle of the insert’s X-axis were expressed as the differences between the angles at full knee extension and the angles at maximum knee flexion.

Regarding the reliability of our 3D-to-2D image registration technique, intraobserver and interobserver reliabilities were examined via intra-class correlation coefficient (ICC) in a previous study. The ICCs (1, 2) of the rotational angle of the axis, AP translation of the medial end of the axis, and AP translation of the lateral end of the axis were 0.98, 0.91, and 0.85, respectively. The ICCs (2, 1) of the rotational angle of the axis, AP translation of the medial end of the axis, and AP translation of the lateral end of the axis were 0.92, 0.86, and 0.99, respectively [27].

### Statistical analysis

All data are expressed as mean ± standard deviation. Shapiro–Wilk analysis was performed to test for normally distributed variables. Paired *t-*tests were used to analyze differences in the AP translation between the medial and lateral most distal points of the femoral component as well as differences in the changes in the rotational angle between the FCX and X-axis of the insert on the tibial component’s axial plane. A *p*-value of < 0.05 was considered statistically significant. Statistical analysis was performed using the Statistical Package for the Social Sciences software (IBM SPSS Statistics, Version 27.0, IBM Corp., Armonk, NY, USA).

## Results

### AP translations of the femoral component’s most distal points (estimated contact point)

**Fig. 6:**
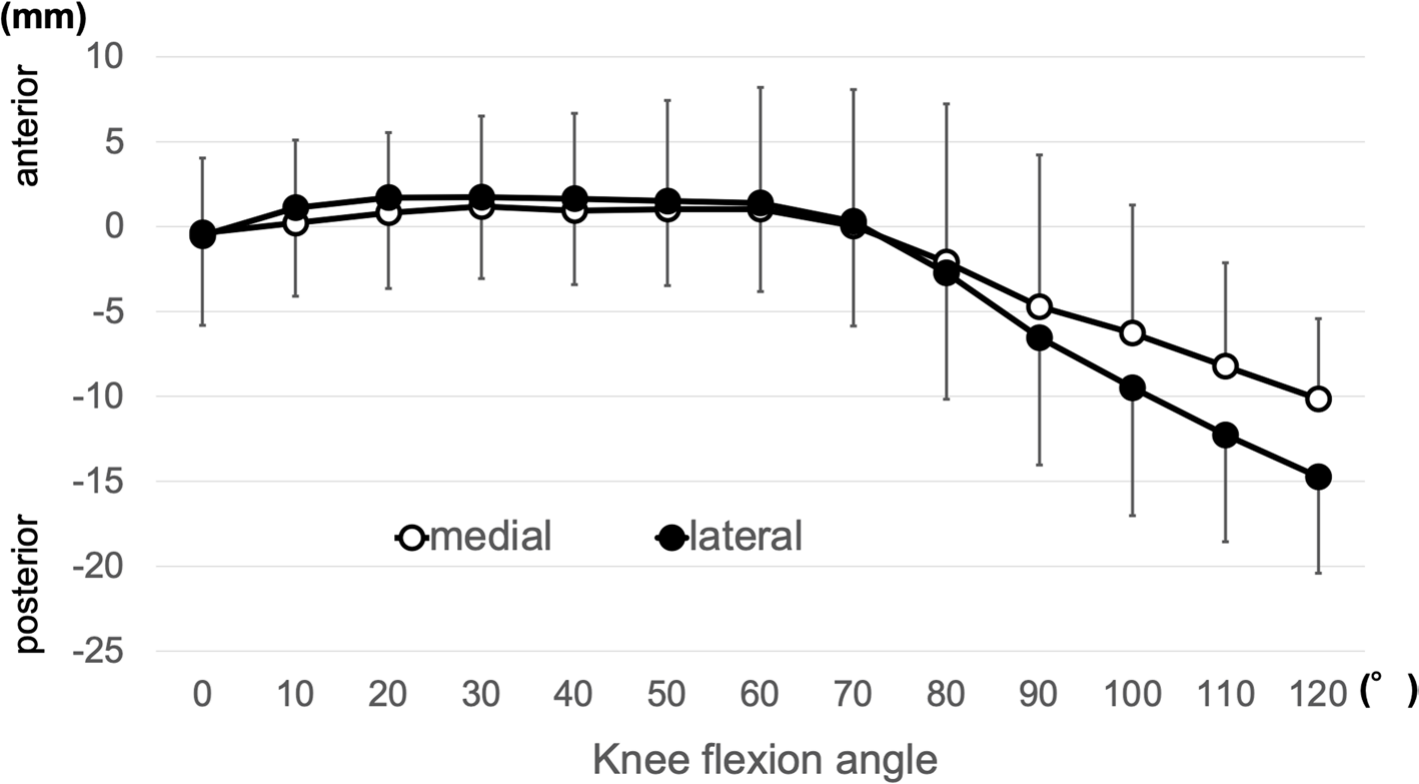
AP translation of the femoral component’s medial and lateral most distal points (estimated contact point) (mean ± SD)

Values in the range of the knee flexion angle (0°–120°) that could be obtained by all subjects were employed for calculations. The femoral component’s medial most distal point showed no marked AP translation until approximately 60° and then showed a gradual posterior translation (mean translation, 8.4 ± 2.5 mm; range, 4.9–11.8 mm posterior). The lateral side also demonstrated no marked AP translation until approximately 60° and then showed a gradual posterior translation (mean translation, 13.6 ± 3.3 mm; range, 9.5–17.7 mm posterior) (Fig. 6). The femoral component showed a bicondylar rollback pattern in which both medial and lateral ends moved posteriorly after 60°. A medial pivot pattern was observed in cases with a certain amount of posterior translation difference between the medial and lateral ends (medial < lateral). The amount of posterior translation of the lateral side was greater than that of the medial side in all cases. The AP translation of the femoral component’s medial most distal point was significantly smaller than that of the lateral point (*p* = 0.001).

### Changes in the FCX angle on the tibial component’s axial plane (rotational angle)

**Fig. 7:**
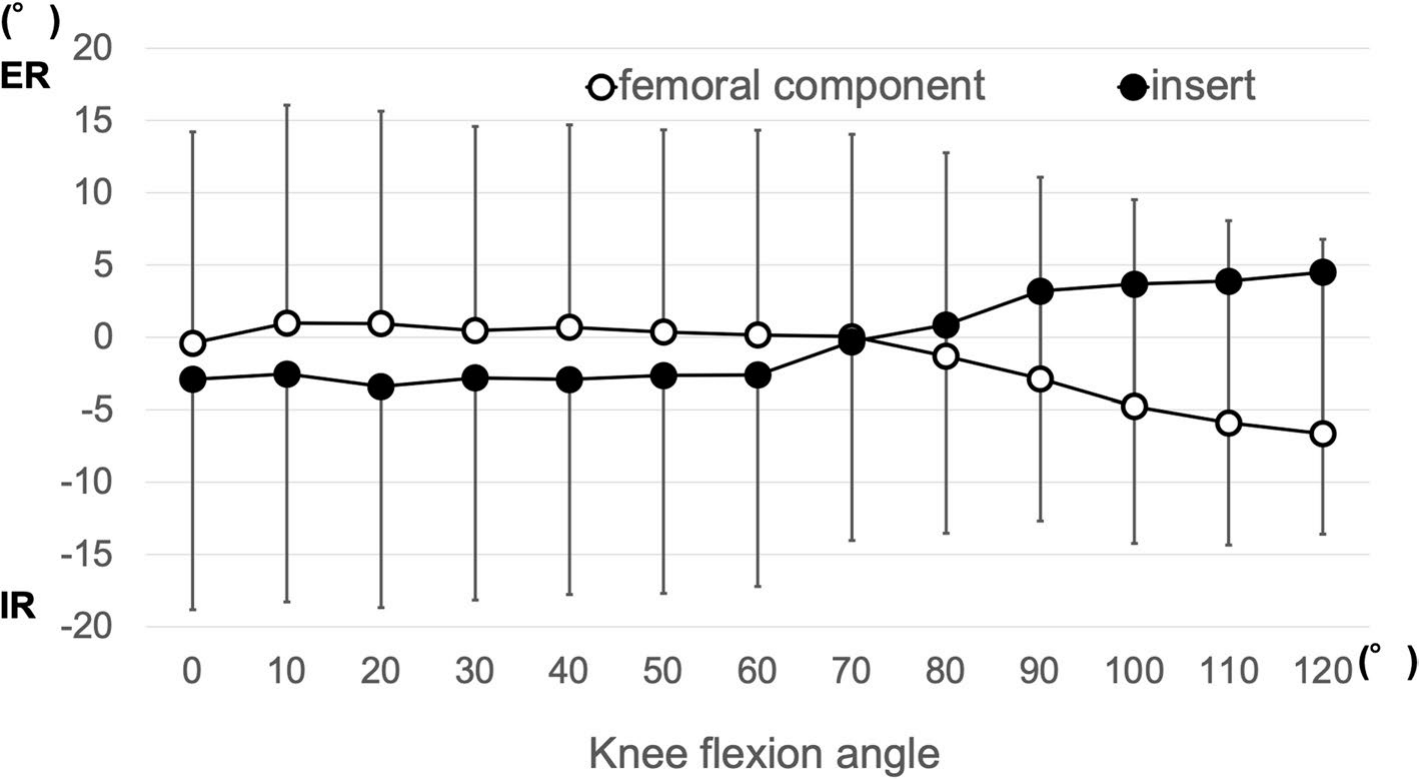
Rotational angle of the FCX and insert’s X-axis (mean ± SD). ER: external rotation, IR: internal rotation

The FCX exhibited external rotation on the axial plane of the tibial component’s coordinate system throughout knee flexion in all subjects, indicating that all subjects demonstrated the internal rotation of the tibial component relative to the femoral component throughout knee flexion. The mean ± standard deviation (SD) rotational angle was 10.7° ± 4.9° external rotation (range, 5.4°–16.4° external rotation) (Table 1). The FCX angles in the knee flexion angle range (0°–120°) that could be obtained for all subjects are shown in Fig. 7.

**Table 1:** The rotational angle of the femoral component and the insert relative to the tibial component

	Femoral component (°)	Insert (°)
Case 1	9.6	-10.0
Case 2	16.4	-12.0
Case 3	15.1	-8.5
Case 4	7.0	-2.0
Case 5	5.4	-12.0
mean	10.7 ± 4.9	-8.9 ± 4.1

Values are presented as absolute value only or mean ± standard deviation (°)

Plus values show external rotation

### Changes in the angle of the insert’s X-axis on the tibial component’s axial plane (motion pattern of the insert)

The mobile insert exhibited internal rotation on the axial plane of the tibial component’s coordinate system throughout knee flexion in all subjects, indicating that all subjects demonstrated opposite patterns relative to the femoral component. In three of the five cases, the lateral side of the inserts exhibited an anterior overhang in the deep flexion phase. The mean ± SD rotational angle was 8.9° ± 4.1° internal rotation (range, 2.0°–12.0° internal rotation) (Table 1). Changes in the angle of the insert’s X-axis were significantly more internally rotated than those of the femoral component’s axis (*p* = 0.004). The angles of the insert’s X-axis in the knee flexion angle range (0°–120°) that could be obtained for all subjects are shown in Fig. 7.

## Discussion

The important findings of the present study were that the amount of posterior translation of the lateral side of the femoral component was greater than that of the medial side in all cases. Hence, a medial pivot pattern was identified. Moreover, the femoral component exhibited external rotation throughout knee flexion in all subjects, whereas the mobile insert exhibited internal rotation (opposite pattern relative to the femoral component).

Regarding the AP translations of the femoral component in our study, no marked AP translation was observed until approximately 60°, and a gradual posterior translation was observed after 60° in the medial and lateral most distal points. A posterior translation of 8.4 mm was detected in the medial most distal point, whereas a posterior translation of 13.6 mm was detected in the lateral most distal point. A previous in vivo tibiofemoral kinematic analysis of TKA with a ball and socket joint geometry at the medial side showed similar results; this study showed less AP translation on the medial side (1.2 mm posteriorly) compared with that on the lateral side (7.3 mm posteriorly) using a 3D-to-2D image matching technique [18]. On the other hand, medial paradoxical anterior translation has been reported in posterior-stabilized (PS) and cruciate-retaining (CR) TKAs with low conformity surface [5, 8, 14]. A recent study demonstrated that paradoxical anterior translation can be reduced by increasing medial conformity of the bearing insert in PS TKA [13]. These studies combined with the results of the present study indicate that high medial conformity may contribute to the suppression of paradoxical anterior translation.

Regarding the rotation of the femoral component, a medial pivot pattern was observed in cases with a certain amount of difference in the posterior translation between the medial and lateral sides (medial < lateral). Some studies reported that TKA with highly medially constrained inserts showed a medial pivot motion [18–20]. Omori et al. evaluated the tibiofemoral contact kinematics of TKA with a ball and socket joint geometry at the medial side using a cadaveric knee and showed medial pivot motion [20]. Miyazaki et al. conducted in vivo tibiofemoral kinematic analysis of TKA with a ball and socket joint geometry at the medial side using a 3D-to-2D image matching technique and showed medial pivot motion [18]. Meanwhile, some studies reported that MBTKA with a flat surface insert does not show medial pivot motion [15, 31]. These results suggest that the highly constrained surface shape of the medial side of the insert is significantly related to medial pivot motion.

Regarding the rotation of the insert, some studies reported that the femoral component and insert showed the same rotation in the motion analysis of TKA with a mobile-bearing mechanism in the motion pattern of the insert [10, 33]. Futai et al. performed an in vivo kinematic analysis of MBTKA during deep-knee bending under weight-bearing conditions and showed that the amounts of external rotation of the femoral component relative to the tibial component and insert relative to the tibial component were 10.4° and 10.2°, respectively, for 0°–120° of flexion. On average, the femoral component showed slight rotation relative to the insert [10]. Yamazaki et al. also conducted an in vivo kinematic analysis of MBTKA and revealed that the femoral component rotated externally with respect to the tibial component during flexion, with an average external rotation range of 12.4°. Similarly, the mobile-bearing insert rotated externally with respect to the tibial component during flexion, with an average rotation range of 11.5°. The axial rotations of the femoral component and mobile-bearing insert with respect to the tibial component were also similar, showing no significant difference. The movement for axial rotation of the femoral component with respect to the mobile-bearing insert exhibited little rotation [33]. The mobile-bearing inserts utilized in these studies had a symmetrical medial and lateral surface. Meanwhile, motion analysis of TKA was performed in this study using mobile-bearing inserts with high medial constraint and asymmetrical medial and lateral surfaces. As a result, the femoral component and mobile-bearing insert showed opposite rotation during knee flexion.

**Fig. 8:**
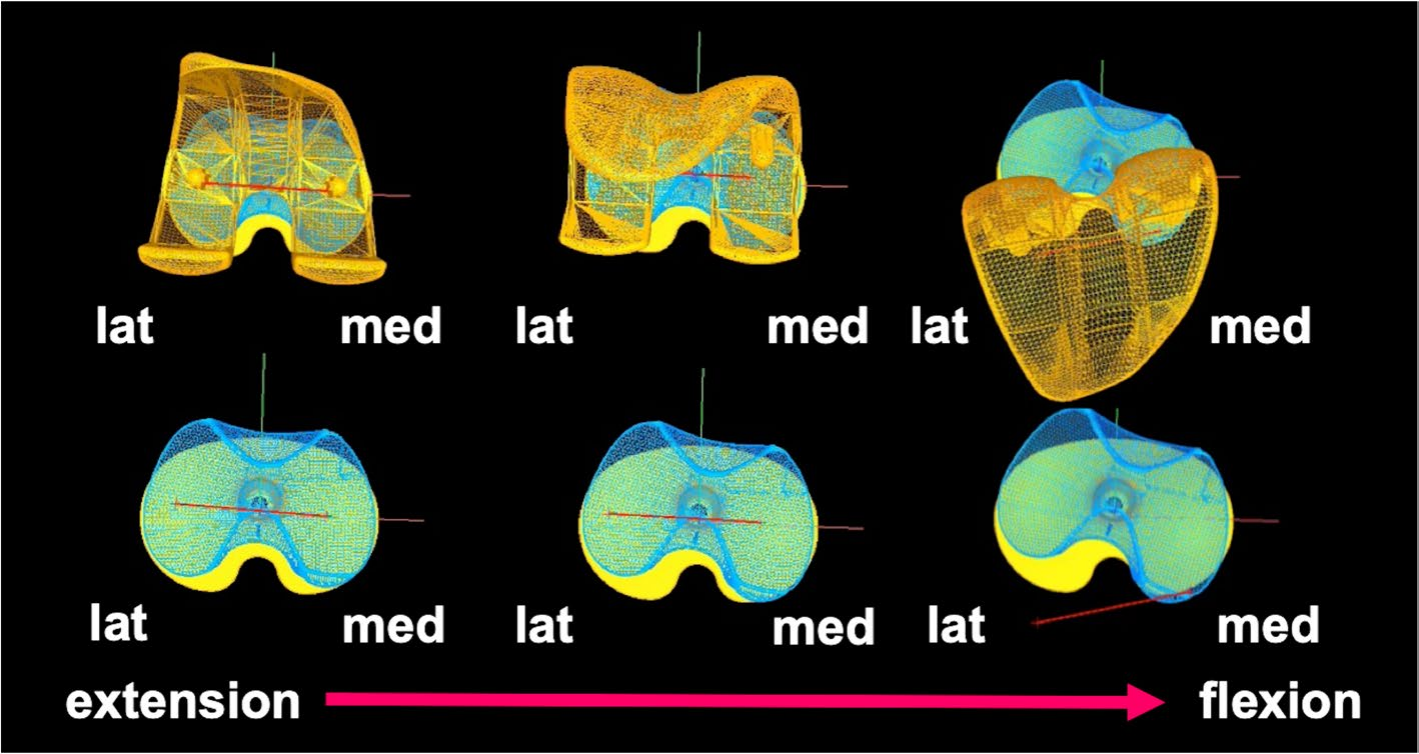
Motion patterns of the femoral component and insert are shown. The femoral component exhibited external rotation throughout knee flexion. Meanwhile, the mobile insert exhibited internal rotation throughout knee flexion. These kinematics were due to the high conformity only on the medial side and less on the lateral side

In normal knees, the amount of AP translation of the femoral condyle is lower on the medial side than that on the lateral side, and the femur exhibits external rotation relative to the tibia (medial pivot motion) and subsequent bicondylar rollback in the late flexion phase [26, 27]. Based on the superior patient-reported outcome measures for MPTKA, reproducing native kinematics is an important factor in improving patient satisfaction [9, 12, 22]. In this study, MMPTKA exhibited medial pivot motion. Moreover, the medial side of the femoral component moved posteriorly with the insert in the late flexion phase due to high conformity only on the medial side, whereas the less constrained lateral side of the femoral component moved posteriorly without being constrained by the anterior movement of the insert (Fig. 8). Thus, femoral components exhibited bicondylar rollback motion in the late flexion phase. In other words, the kinematics between the femoral component and tibial component were ideal and in line with the design concept.

All inserts in this study showed internal rotation throughout knee flexion. However, the rotational angles differed from each other. This may be due to the differences in soft-tissue tightness and component position. In addition, the lateral side of the inserts showed an overhang in the deep flexion phase, and the effect of this phenomenon remains a clinical concern. Kinematic changes in the mobile-bearing insert over time in patients are also unclear. The rotation of the mobile-bearing insert has been reported to decrease over time [32]. However, other studies have reported that the rotation of the mobile-bearing insert is maintained over time [7, 34]. Kinematic changes in the mobile-bearing insert may require further investigation, especially with the effects of soft tissue balance on in vivo motion, although these components have already been clinically used in Europe and Japan and no major complications related to the inserts in the short- and mid-term have been detected.

This study had several limitations. First, this cadaveric study had a small sample size. Although the same trends were observed in all five samples, it is better to include a larger sample size. Second, quantitative evaluations of soft tissue balancing and postoperative component positions were difficult. Therefore, in vivo studies that allow these evaluations are desirable in the future.

Regarding clinical relevance, the results of this study provide valuable kinematic information about MMPTKA that has not been clear yet and may be used as a reference for in vivo motion analyses.

## Conclusions

The posterior translation of the lateral side of the femoral component was greater than that of the medial side in all cases. Hence, a medial pivot pattern was identified. The femoral component exhibited external rotation throughout knee flexion in all subjects, whereas the mobile insert exhibited internal rotation (opposite pattern relative to the femoral component). This study provides valuable kinematic information on MMPTKA that has not been clear yet.

## Data Availability

The datasets analyzed in this study are available from the corresponding author on reasonable request.

## Abbreviations

TKA: Total knee arthroplasty
OA: Osteoarthritis
MBTKA: Mobile-bearing-type TKA
MPTKA: Medial pivot-type TKA
MMPTKA: Mobile medial pivot-type TKA
3D: Three-dimensional
CT: Computed tomography
AP: Anteroposterior
FCX: Femoral component’s X-axis
ICC: Intra-class correlation coefficient
SD: Standard deviation
